# Prevalence, awareness and control of diabetes in Russia: The Ural Eye and Medical Study on adults aged 40+ years

**DOI:** 10.1371/journal.pone.0215636

**Published:** 2019-04-22

**Authors:** Mukharram M. Bikbov, Rinat R. Fayzrakhmanov, Gyulli M. Kazakbaeva, Rinat M. Zainullin, Inga I. Arslangareeva, Timur R. Gilmanshin, Venera F. Salavatova, Nikolai A. Nikitin, Svetlana R. Mukhamadieva, Dilya F. Yakupova, Renat I. Khikmatullin, Artur F. Zaynetdinov, Yulia V. Uzianbaeva, Said K. Aminev, Ildar F. Nuriev, Jost B. Jonas

**Affiliations:** 1 Ufa Eye Research Institute, Ufa, Bashkortostan, Russia; 2 Department of Ophthalmology, Medical Faculty Mannheim of the Ruprecht-Karls-University of Heidelberg, Mannheim, Germany; Public Library of Science, UNITED KINGDOM

## Abstract

**Purpose:**

Non-communicable chronic diseases have become the leading causes of mortality and disease burden worldwide. With information about the frequency of diabetes as a major non-communicable chronic disease in Russia being scarce, we assessed the prevalence of diabetes and its associated factors in a rural and urban population in Russia.

**Methods:**

The Ural Eye and Medical Study is a population-based study in the city of Ufa/Russia and in villages in a distance of 65 km from Ufa. Inclusion criterion was an age of 40+ years. All study participants underwent a standardized interview and a detailed general examination. Diabetes mellitus was defined by a plasma glucose concentration ≥7.0 mmol/L or self-reported history of physician diagnosis of diabetes.

**Results:**

Out of a population of 7328 eligible individuals, 5899 individuals (2580 (43.7%) men) (participation rate:80.5%) participated (mean age:59.0±10.7 years (range:40–94 years)). Diabetes mellitus was present in 687 individuals (11.7%;95% confidence interval (CI):11.9,12.5). Awareness rate of having diabetes was 500/687 (72.8%;95%CI:69.0,76.0), with mean known duration of diabetes of 10.0±9.4 years. Known type 1 diabetes was present in 44 subjects and known type 2 diabetes in 358 subjects. Prevalence of undiagnosed diabetes was 3.2% (95%CI:2.7,3.6) in the study population. Among patients with diabetes, 59.1% (95%CI:55.4,62.8) received treatment for diabetes, among whom 237 (58.5%;95%CI:53.7,63.3) individuals had adequate glycemic control. In multivariable analysis, higher prevalence of diabetes mellitus was associated with older age (*P*<0.001; odds ratio (OR):1.03;95%CI:1.01,1.04), higher body mass index (*P*<0.001;OR:1.07;95%CI:1.04,1.10), lower prevalence of vigorous daily work (*P* = 0.002;OR0.68;95%CI:0.53,0.87), positive history of arterial hypertension (*P* = 0.03;OR:1.40;95%CI:1.03,1.89) and cardiovascular diseases (*P* = 0.001;OR:1.60;95%CI:1.21,2.13) including heart attacks (*P* = 0.01;OR:1.80;95%CI:1.15,2.81), higher serum concentration of triglycerides (*P*<0.001;OR:1.51;95%CI:1.30,1.75), higher systolic blood pressure (*P* = 0.01;OR:1.01;95%CI:1.01,1.02), higher number of meals taken daily (*P*<0.001;OR:1.46;95%CI:1.25,1.69), and non-Muslim religion (*P* = 0.02;OR:0.73;95%CI:0.56,0.94).

**Conclusions:**

In this ethnically mixed, urban and rural Russian population aged 40+ years, the awareness rate of diabetes (72.8%) was relatively high, while the diabetes prevalence (11.7%) was comparable with that of other countries such as China and the USA. Factors associated with higher diabetes prevalence were similar in Russia and these other countries and included older age, higher body mass index and higher serum concentration of triglycerides, lower prevalence of vigorous daily work, arterial hypertension and cardiovascular diseases.

## Introduction

Diabetes mellitus has become one of the most important causes of disability and death in all world regions. In the recent Global Burden of Diseases Study 2015, diabetes has climbed from position # 24 in 1990 through position #16 in 2005 to position #11 in 2015 in the ranking list of the most frequent causes of global DALYs (disability-adjusted life-years) for both sexes [1]. In a parallel manner, diabetes besides ischemic heart disease and stroke was among the leading causes of years of life lost (YLLs) in most world regions [2]. Despite the high importance of diabetes for public health and although Russia is by area the largest, and by population one of the most populous, countries worldwide, information about the prevalence of diabetes in Russia and factors associated with the occurrence of diabetes in Russia has remained scarce [3–5]. A large-scaled investigation on 26,620 adult Russians assessed the prevalence of diabetes, however, the recruitment occurred in highly frequented public areas and it was not purely population-based [4]. Another Russian investigation on the prevalence of diabetes was performed in a selected relatively small study population in the Siberian region of Krasnoyarsk [5]. Other studies examined the frequency of an impaired fasting glucose and the prevalence of the metabolic syndrome and on familial hypercholesterolemia in relatively small cohorts of Yakut adults in North-West Siberia [6,7]. In view of the scarcity of data on the prevalence of diabetes and its associated factors available for Russia and in view of the importance of diabetes, we therefore conducted this study to assess the prevalence of diabetes in a population in Russia and to explore associations between diabetes and other major risk factors. Since Russia includes many ethnicities, we chose as study area a region with a population consisting of Russians and other ethnic groups with different cultural background.

## Methods

The Ural Eye and Medical Study (UEMS) is a population-based cross-sectional study performed in the urban region of Kirovskii in the city of Ufa and in villages of the rural region of the Karmaskalinsky District in a distance of 65 km from Ufa in direction to the Ural Mountains. It was conducted from 2015 to 2017. According to the Declaration of Helsinki, the Ethics Committee of the Academic Council of the Ufa Eye Research Institute approved the study and all participants gave informed written consent. The ethics committee confirmed that all methods were performed in accordance with the relevant guidelines and regulations. Inclusion criterion for the participation in the study was an age of 40+ years, with no exclusion criteria. The reasons to choose an age of 40+ years as inclusion criterion was that the prevalence of major disorders starts to increase beyond that age, so that with that age value the study population would include a sufficient number of patients affected by the disease, and a sufficient number of healthy control individuals. In addition, many population-based studies have chosen this age-limit as inclusion criterion, so that the results of the present study would easier to be compared with the observations made in previous investigations. The study has been described in detail previously [8–10]

All study participants underwent an interview by trained social workers using a standardized questionnaire with more than 250 questions on socioeconomic parameters such as level of education and living conditions, diet, smoking or other types of tobacco consumption, daily physical activity, alcohol consumption, depression and suicidal ideas, and medical history including known diagnosis and therapy of major diseases such as arterial hypertension, diabetes mellitus, and cardiovascular diseases (Table 1). Most of the questions included in the questionnaire were part of standardized questionnaires such as the Zung self-rated depression scale and the Mini Mental Status Examination test [11,12]. For the assessment of smoking, the questions required information on current or former smoking, daily smoking, age when smoking started, package years of smoking, use of smokeless tobacco, when the first cigarette is smoked after awakening, smoking in smoking-free places, and smoking when ill. For the assessment of alcohol consumption, we asked about current and former drinking, its quantity and type of beverages drunk, age when drinking started (and stopped if at all), inability to stop when the first drink has been taken, failure of performance due to drinking and need of a first drink in the morning. For all subjects, we measured arterial blood pressure, pulse rate, body height, body weight and circumference of the hip and waist, and we performed biochemical examinations of blood samples taken under fasting conditions. The body height was determined in a standardized manner with the shoes routinely removed. The subjects were asked to stand upright as much as possible and with the head raised upright as much as possible. We used a stadiometer as measuring instrument. The floor was completely even. We did not take into account, nor did we correct for age-related reductions in height of subjects, who reportedly were taller during their middle-age. The handgrip strength was measured using a dynamometer (dynamometer-dk 140, ZAO Nizhnetagilsk Medical Instrument Plant, Nizhny Tagil, Russia). Hearing loss was examined performing Rinne´s test and Weber´s test (c2 512 Hz; Kirchner & Wilhelm Gmbh & Co, Asperg, Germany). Pulmonary function was tested by spirometric measurement of the forced expiratory volume in one second (Riester spirotest, Riester Company, Jungingen, Germany). Arterial hypertension was defined as a systolic blood pressure ≥140 mmHg and/or a diastolic blood pressure ≥90 mmHg, and/or self-reported history or current treatment of arterial hypertension with antihypertensive medication. To assess depression, we used the Center for Epidemiologic Studies Depression Scale (CES-D) Scoresheet. The State-Trait Anxiety Inventory (STAI) was used to assess trait and state anxiety.

**Table 1:** Demographic, socioeconomic, lifestyle-associated, and other parameters (mean ± standard deviations; frequency and 95% confidence intervals) in the Ural Eye ad Medical Study.

Parameter	Non-Diabetic Group	Diabetic Group	*P*-Value
N	5178	687	
Age (years)	58.3 ± 10.6	63.7 ± 9.8	<0.001
Men / women	2305 / 2873 (44.5% / 55.5%)	254 / 433 (37.0% / 63.0%)	<0.001
Urban / rural region of habitation	2091 / 3087 (40.4% / 59.6%)	381 / 306 (55.5% / 44.5%)	<0.001
Family status: Married / Unmarried / Divorced / Widowed / Missing	3841 / 316 / 295 / 724 / 2	446 / 58 / 43 / 139 / 1	<0.001
Family status: Married versus any other status	3841 / 5178 74.2% (73.0, 75.4)	446 /687 (65.0% (61.4, 68.6))	<0.001
Religion: Muslim / Christian / Other	3272 / 1814 / 92	380 / 296 / 11	<0.001
Ethnicity: Russian / any other ethnicity	1007 / 4783 (21.1% (19.9, 22.2))	176 / 594 (29.6% (26.0, 33.3))	<0.001
Body height (cm)	165.0 ± 8.7	163.5 ± 9.1	<0.001
Body weight (kg)	75.2 ± 14.3	80.9 ± 15.8	<0.001
Body mass index (kg/m^2^)	27.6 ± 4.9	30.3 ± 5.4	<0.001
Waist circumference (cm)	93.2 ± 13.1	100.3 ± 13.7	<0.001
Hip circumference cm)	107.7 ± 13.2	103.1 ± 12.4	<0.001
Level of education	5.9 ± 1.7	5.6 ± 1.8	0.001
How long is your usual work day? (Minutes)	462 ± 263	406 ± 245	<0.001
Does your work involve physically vigorous activity (like heavy lifting or digging) or physically moderate intensity activity (like brisk walking or carrying light loads) (No / Yes)	0.55 ± 0.50	0.42 ± 0.49	<0.001
Per mean day including all days of the week, how much time do you spend with physically moderate to intensive activities as part of your work?	324 ± 235	297 ± 226	<0.001
Over the past 7 days, how much time did you spend sitting or reclining on a typical day?	1060 ± 919	1114 ± 940	<0.001
Vegetarian diet / mixed diet	10 / 5178 (0.2% (0.2, 0.2))	0 / 687 (0%)	0.62
Do you currently smoke any tobacco products? (yes)	686 / 5171 (13.3% (12.3, 14.2))	51 / 687 (7.4% (5.5, 9.4))	<0.001
Smoking package years (package = 20 cigarettes)	4.3 ± 13.0	2.5 ± 10.2	<0.001
Alcohol consumed such as beer, whisky, rum, gin brandy or other local products? (yes / no)	1129 / 5178 (21.8% (20.7, 22.9))	116 / 687 (16.9% (14.1, 19.7))	0.003
Forced vital capacity	2238 ± 540	2076 ± 538	<0.001
Forced expiratory volume in one second	1914 ± 473	1784 ± 489	<0.001
Vital capacity	2543 ± 598	2363 ± 596	<0.001
Tidal volume	533 ± 148	474 ± 135	<0.001
Tiffno index	75.1 ± 5.8	75.0 ± 6.1	0.73
Gaenslar index	55.6 ± 23.6	52.8 ± 22.7	0.004
Depression score (adapted)	1.11 ± 3.72	1.71 ± 3.90	<0.001
State-Trait Anxiety Inventory (STAI) Score (adapted)	-0.72 ± 3.52	-0.21 ± 3.64	0.001
Manual dynamometry, right hand (dekaNewton)	30.9 ± 11.7	27.4. ± 11.5	<0.001

Diabetes mellitus was defined by a fasting blood glucose concentration ≥7.0 mmol/L or by a self-reported history of physician diagnosis of diabetes mellitus or by a history of drug treatment for diabetes (insulin or oral hypoglycemic agents). The measurement of the fasting blood glucose concentration was performed for all study participants. Awareness of diabetes was defined as self-report of any prior diagnosis of diabetes by a health care professional.

Statistical analysis was performed by using a commercially available statistical software program (Statistical Package for Social Science, SPSS, version 23.0; IBM-SPSS Inc., Chicago, USA). We first calculated the prevalence of diabetes and presented the results as mean and 95% confidence intervals (CI). In a univariate analysis, we assessed associations between the prevalence of diabetes and other parameters such as age and body mass index. Finally, we performed a multivariable binary regression analysis which included the prevalence of diabetes as dependent variable and as independent variables all those parameters which were significantly correlated with the diabetes prevalence in the univariate analysis. We calculated odds ratios (OR) and their 95% confidence intervals (CI). All *P-*values were two-sided and considered statistically significant when the values were less than 0.05.

## Results

The study included 5899 individuals (2580 (43.7%) men) out of a population of 7328 eligible individuals, with a participation rate of 80.5%. The non-participation of the eligible but non-participating individuals was due to the individuals´ decision not to participate. The mean age of the study population was 59.0 ± 10.7 years (median: 58 years; range: 40–94 years). The composition of the study population with respect to gender and age corresponded to the gender and age distribution in the Russian population according to the census carried out in 2010, with no significant difference between both populations (*P* = 0.25) (Table 2) [13]. With respect to the ethnic composition, the percentage of the non-Russian groups was higher in the present study population than in the population of all Russia. For that reason, the statistical analysis was additionally performed separately for both groups, the Russian group and the non-Russian group. Mean body height was 164.8 ± 8.8 cm (median: 164 cm; range: 112–196 cm), mean body weight was 75.9 ± 14.6 kg (median: 75 kg; range: 31–170 kg), and mean body mass index was 27.9 ± 5.0 kg/m^2^ (median: 27.4 kg/m^2^; range: 13.96–60.96 kg/m^2^). Illiteracy (equivalent to level 0 or pre-primary education of the International Standard Classification of Education (ISCED) was present for 17 (0.3%) individuals, 104 (1.8%) participants had passed the fifth grade (equivalent to ISCED level I; primary education or first stage of basic education), 593 (10.1%) participants the 8th grade (equivalent to ISCED level II or lower secondary education), 659 (11.2%) participants the 10th grade (equivalent to ISCED level III or upper secondary education), and 782 (13.3%) individuals the 11th grade (equivalent to ISCED level IV or post-secondary non-tertiary education). Graduates (equivalent to ISCED level V or first stage of tertiary education) were 2052 (34.8%) individuals, and post graduates (equivalent to ISCED level VI or second stage of tertiary education) were 52 (0.9%) study participants. A specialized secondary education had been achieved by 1638 (27.8%) individuals.

**Table 2:** Fasting blood concentrations of glucose in the Ural Eye and Medical Study stratified by age and gender.

Age Group (Years)	n	Percentage of the whole study population (%)	Percentage of the Russian population aged 40+ years(%)	Fasting Blood Concentration of Glucose (mmol/L) (mean ± standard deviation
Men				
40–44	213	8.26	17,95	4.81 ± 1.13
45–49	355	13.76	14,87	4.95 ± 1.59
50–54	437	16.94	15,90	4.80 ±1.44
55–59	482	18.68	16,92	4.95 ±1.59
60–64	403	15.62	14,87	4.97 ±1.49
65–69	292	11.32	9,74	5.13 ± 1.80
70–74	137	5.31	5,13	5.23 ± 1.48
75–79	164	6.36	4,62	5.26 ± 2.31
80+	76	2.95	4,62	5.49 ± 2.31
Women				
40–44	281	8.47	13,21	4.62 ± 0.94
45–49	387	11.66	11,43	4.63 ± 1.01
50–54	491	14.79	13,21	4.85 ± 1.38
55–59	549	16.54	15,00	5.12 ± 1.83
60–64	513	15.46	14,29	5.28 ± 2.05
65–69	496	14.94	10,36	5.27 ± 1.83
70–74	221	6.66	6,43	5.41 ± 2.17
75–79	245	7.38	7,14	5.30 ±1.76
80+	123	3.71	8,93	5.32 ± 1.70

Measurements of the blood concentration of glucose and data on the history of known diabetes were available for 5865 (99.4%) individuals out of the whole study population. The individuals participating in the present investigation and the subjects without valid blood glucose measurements did not differ in age (59.0 ± 10.7 years versus 60.6 ± 13.0 years; *P* = 0.47), systolic blood pressure (133.5 ± 20.0 mmHg versus 133.9 ± 20.0 mmHg; *P* = 0.43) and diastolic blood pressure (82.0 ± 10.4 mmHg versus 80.2 ± 12.6 mmHg; *P* = 0.41). Both groups differed significantly in gender (2559 men / 3306 women versus 21 men / 13 women; *P* = 0.38) and in region of habitation (2472 city / 3393 village versus 27 city / 7 village).

Mean serum concentration of glucose was 5.03 ± 1.67 mmol/L (median: 4.8 mmol/L; range: 1.95–22.5 mmol/L). A glucose serum concentration of ≥7 mmol/L was present in 389 (6.63%; 95%CI: 6.00, 7.27) study participants (Table 2). Known diabetes mellitus was present in 500 (8.5% of the total study population) individuals with a mean age of 64.4 ± 9.3 years (range: 40–88 years) (Table 3). Insulin therapy was used by 70 participants (14.0% of the patients with diabetes and 1.2% of the whole study population), oral diabetic therapy was performed in 306 participants (61.3% of the patients with diabetes), and 30 participants (6.0% of the whole study population) with known diabetes were only on anti-diabetic diet. For 94 (18.6%) individuals with self-reported diabetes, no therapy or diet was reported. Combining these data, diabetes mellitus was present in 687 individuals (11.7%; 95%CI: 11.89, 12.53). The awareness rate of having diabetes was 500 / 687 or 72.8% (95%CI: 69.0, 76.0). For 327 individuals who could remember when their diabetes was detected, known duration of diabetes was (10.0 ± 9.4 years (median: 7 years; range: 1 to 71 years). For 173 diabetic individuals, the known duration of diabetes could not be well remembered by the participants. Out of the 500 individuals with known diabetes, 44 subjects (or 10.9% out of 402 diabetic individuals with knowledge about the type of their diabetes) reported to have type 1 diabetes and 358 subjects (or 71.6% out of 402 diabetic individuals with knowledge about the type of their diabetes) indicated to have type 2 diabetes, while 98 (98/500 or 19.6%) did not know the type of their diabetes. The prevalence of undiagnosed diabetes was 3.2% (95% CI: 2.7, 3.6): 3.6% (95% CI: 2.9, 4.4) in men and 2.8% (95% CI: 2.3, 3.4) in women. Among the diabetic patients with anti-diabetic therapy (n = 406 individuals or 59.1% (95% CI: 55.4, 62.8) of all diabetic patients), 237 (58.5% (95%CI: 53.7, 63.3) individuals had a blood glucose concentration of lower than 7 mmol/L and were thus considered to have adequate glycemic control.

**Table 3:** Prevalence of diabetes mellitus in the Ural Eye and Medical Study stratified by age and gender.

Age Group (Years)	Diabetes Prevalence	95% Confidence Intervals
Men		
40–44	5.16	2.17, 8.16
45–49	5.92	3.45, 8.38
50–54	5.95	3.72, 8.18
55–59	10.58	7.83, 13.34
60–64	9.68	6.78, 12.58
65–69	14.73	10.64, 18.81
70–74	15.33	9.22, 21.44
75–79	18.29	12.31, 24.27
80+	15.79	7.40, 24.18
Women		
40–44	2.14	0.43, 3.84
45–49	5.17	2.95, 7.38
50–54	6.31	4.15, 8.47
55–59	11.48	8.80, 14.15
60–64	16.76	13.52, 20.01
65–69	21.57	17.94, 25.20
70–74	21.27	15.83, 26.70
75–79	20.41	15.33, 25.49
80+	18.70	11.71, 25.69

Within the Russian ethnic group (n = 1183; 506 men, 677 women) with a mean age of 60.1 ± 11.1 years, diabetes mellitus was present in 176 out of 1183 individuals (14.9% (95%CI: 12.9, 16.9) with an awareness rate of 140/176 or 79.5% (95%CI: 73.5, 85.5) and a known duration of diabetes of 11.7 ± 11.5 years (median: 9 years; range: 1 to 71 years). Out of the 140 individuals being aware of their disease, 14 (10.0%) reported to have type 1 diabetes and 110 (78.6%) subjects indicated to have type 2 diabetes, while 16 (11.4%) did not know the type of their diabetes. Within the Russian subgroup, the prevalence of undiagnosed diabetes was 36/176 (20.5%; 95%CI: 14.4, 26.5); for men: 25.9% (95% CI: 14.3, 37.5); for women: 17.8% (95% CI: 10.8, 24.8)). Out of the 140 individuals with known diabetes, 26 subjects (18.6%) were on insulin therapy, 90 (64.3%) on oral therapy, and 9 (6.4%) on diet. Fifteen individuals (10.7%) did not undergo any therapy. Referring to the total group of Russians with diabetes, 125 individuals (71.0% (95% CI: 64.3, 77.8) received treatment for diabetes. Among these 125 diabetic individuals with diabetic therapy, 65 (52.0% (95%CI: 43.1, 60.9) individuals had a blood glucose concentration of lower than 7 mmol/L and were considered to have adequate glycemic control.

Within the non-Russian ethnic group (n = 4194; 1927 men, 2267 women) with a mean age of 58.2 ± 11.4 years, diabetes mellitus was present in 418 out of 4194 individuals (10.0% (95%CI: 9.1, 10.9) with an awareness rate of 304/418 or 72.7% (95%CI: 68.4, 77.0) and a known duration of diabetes of 8.8 ± 7.2 years (median: 7 years; range: 1 to 57 years). Out of the 304 non-Russian individuals being aware of their disease, 29 (9.5%) reported to have type 1 diabetes and 248 (81.6%) subjects indicated to have type 2 diabetes, while 27 (8.6%) did not know the type of their diabetes. Within the non-Russian subgroup, the prevalence of undiagnosed diabetes was 114/418 (27.3%; 95%CI: 23.0, 31.6); for men: 62/172 (36.0%; 95%CI: 28.8, 43.3); for women: 52/246 (21.1%; 95%CI: 16.0, 26.3)). Out of the 304individuals with known diabetes, 44 subjects (14.5%) were on insulin therapy, 216 (71.1%) on oral therapy, and 21 (6.9%) on diet. Twenty-three individuals (7.6%) did not undergo any therapy. Referring to the total group of non-Russians with diabetes, 281 individuals (67.2% (95% CI: 62.7, 71.7) received treatment for diabetes. Among these 281 diabetic individuals with diabetic therapy, 186 (44.5% (95%CI: 39.8, 49.4) individuals had a blood glucose concentration of lower than 7 mmol/L and were considered to have adequate glycemic control.

Comparing the Russian group with the non-Russian group revealed that the diabetes prevalence was significantly (*P*<0.001) higher in the Russian group (14.9% (95%CI: 12.9, 16.9) versus 10.0% (95%CI: 9.1, 10.9), while the awareness rate (*P* = 0.10) and the rate of adequate glycemic control (*P* = 0.08) did not differ markedly between the ethnic groups.

**Fig 1:**
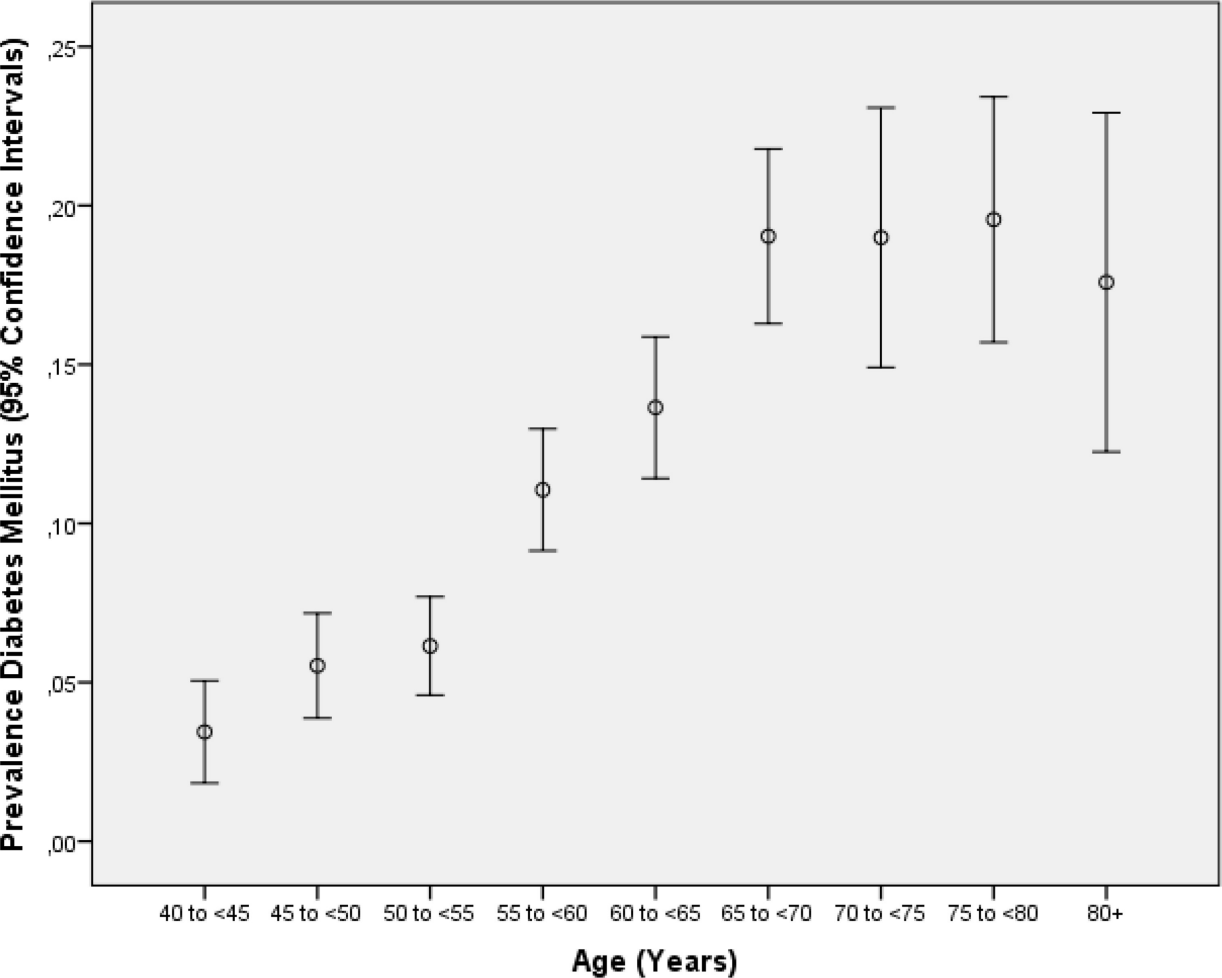
Graph showing the distribution of diabetes mellitus (defined by a plasma glucose concentration ≥7.0 mmol/L or by a self-reported history of physician diagnosis of diabetes mellitus or by a history of drug treatment for diabetes) stratified by age in the Ural Eye and Medical Study.

**Fig 2:**
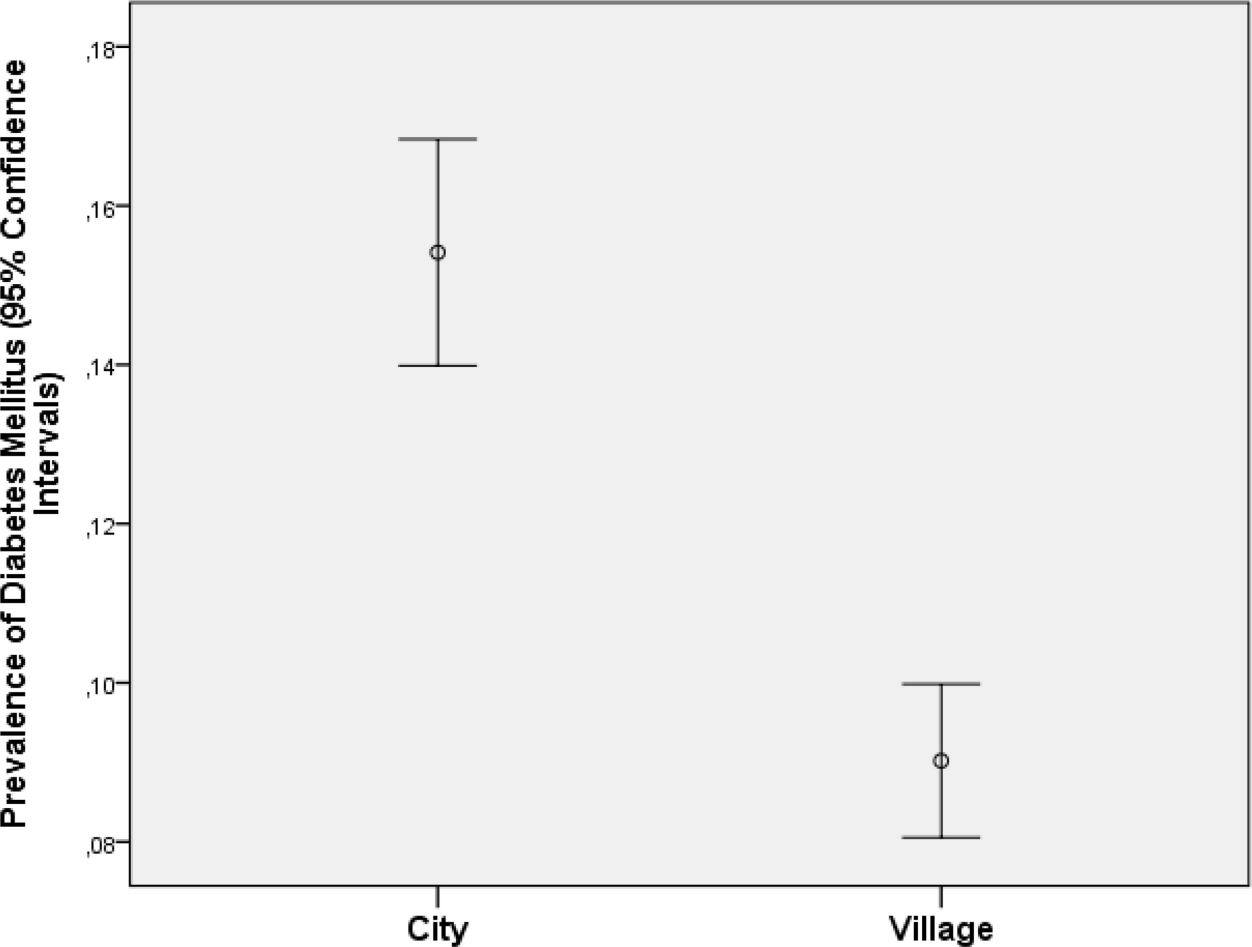
Graph showing the distribution of diabetes mellitus (defined by a plasma glucose concentration ≥7.0 mmol/L or by a self-reported history of physician diagnosis of diabetes mellitus or by a history of drug treatment for diabetes) stratified by region of habitation in the Ural Eye and Medical Study.

In univariate analysis, the diabetic group differed from the non-diabetic group in the demographic parameters of older age (*P*<0.001) (Fig 1), preponderance of women (*P*<0.001), urban region of habitation (*P*<0.001) (Fig 2), and various other parameters (Table 1). In the multivariable binary regression analysis, we assessed associations between diabetes prevalence and the independent parameters in a step-wise manner in groups. Out of the group of demographic and socioeconomic parameters, we first dropped, due to collinearity, the parameters of body weight and hip circumference. Due to a lack of statistical significance, we then dropped step by step the parameters of ethnicity (*P* = 0.72), body height (*P* = 0.64), gender (*P* = 0.66), ownership of a two-wheeler (*P* = 0.56), a computer (*P* = 0.19) and house (*P* = 0.95), religion (*P* = 0.41), level of education (*P* = 0.42), and ownership of a telephone (*P* = 0.20). After adding the physical activity-related parameters to the analysis, we dropped the parameters of time spent on sitting or reclining in the last 7 days (*P* = 0.94), physically moderate physical activity as part of leisure time (*P* = 0.82), physically moderate physical activity as part of daily work (*P* = 0.81), physically vigorous physical activity in leisure time (*P* = 0.80), and walking or use of a bicycle for at least 10 minutes (*P* = 0.59). In the resulting model, family status (married or not married) was no longer significantly (*P* = 0.72) associated with diabetes prevalence, so that it was also dropped. After adding the medical history-related parameters, we dropped the parameters of history of thyreopathy (*P* = 0.97), osteoarthritis (*P* = 0.79), hospital admittance due to low blood pressure (*P* = 0.43), and backache (*P* = 0.46). In a next step, we added the blood parameters, and we dropped step by step the parameters of percentage of basophil granulocytes, serum concentrations of high-density lipoproteins (*P* = 0.95), serum concentration of aspartate aminotransferase (*P* = 0.94), rheumatoid factor (*P* = 0.81), cell count of leukocytes (*P* = 0.69), urea (*P* = 0.64), erythrocyte sedimentation rate (*P* = 0.46), serum concentrations of alanine aminotransferase (*P* = 0.26), and percentage of rod-core granulocytes (*P* = 0.19). We then added the blood pressure-associated parameters to the analysis and dropped the parameters of ankle-brachial index for the right side (*P* = 0.46), history of arterial hypertension (*P* = 0.64), diastolic (*P* = 0.38) and mean (*P* = 0.38) blood pressure, serum concentration of residual nitrogen (*P* = 0.98) and creatinine (*P* = 0.27), region of habitation (*P* = 0.63), body waist circumference (*P* = 0.36), and history of blood lipid therapy (*P* = 0.22) and arthritis (*P* = 0.22). We then added the diet-related parameters to the analysis and dropped the parameters of number of servings of vegetables if vegetables were taken (*P* = 0.60) and number of servings of fruits if fruits were taken (*P* = 0.65). We than added the parameters of smoking and alcohol consumption and dropped the parameters of prevalence of alcohol consumption (*P* = 0.99), prevalence of current smoking (*P* = 0.49) and number of cigarette package years (*P* = 0.67). We finally added the remaining parameters and we dropped dynamometric force of the left hand (*P* = 0.94), subjective hearing loss (*P* = 0.99), the results of Rinne´s test (*P* = 0.76), anxiety score (*P* = 0.77), forced expiratory volume in one second (*P* = 0.64), vital capacity (*P* = 0.32), tidal volume (*P* = 0.36), depression score (*P* = 0.29), forced vital capacity (*P* = 0.20), dynamometric force of the right hand (*P* = 0.45), length of a working day (*P* = 0.27), and whether a doctor was seen in the last 12 months (*P* = 0.13).

If we re-added to the model the parameters of gender (*P* = 0.78), region of habitation (*P* = 0.21), level of education (*P* = 0.24), ethnicity (Russian versus non-Russian) (*P* = 0.19), body height (*P* = 0.65), previous history of cancer (*P* = 0.70), diastolic blood pressure (*P* = 0.20), cigarette package years (*P* = 0.46), current smoking (*P* = 0.75), depression score (*P* = 0.13), and anxiety score (*P* = 0.43), none of these parameters were significantly associated with the prevalence of diabetes mellitus. In contrast, if religion and history of arterial hypertension were re-added, both were significantly correlated so that in the final model, higher prevalence of diabetes mellitus was associated with older age (*P*<0.001), higher body mass index (*P*<0.001), lower prevalence of vigorous daily work (*P* = 0.002), positive history of arterial hypertension (*P* = 0.03) and cardiovascular diseases (*P* = 0.001) including heart attacks (*P* = 0.01), higher serum concentration of triglycerides (*P*<0.001), higher systolic blood pressure (*P* = 0.01), higher number of meals taken daily (*P*<0.001), and non-Muslim religion (*P* = 0.02) (Table 4). If the parameter of a history of menopause was added to the model in women, it was not significantly (*P* = 0.13) associated with the prevalence of diabetes.

**Table 4:** Associations (multivariable analysis) of the prevalence of diabetes in the Ural Eye and Medical Study.

Parameter	*P*-Value	Odds Ratio	95% Confidence Interval
Age (years)	<0.001	1.03	1.01, 1.04
Body mass index (kg/m^2^)	<0.001	1.07	1.04, 1.10
Daily work with vigorous physical activity	0.002	0.68	0.53, 0.87
History of cardiovascular diseases	0.001	1.60	1.21, 2.13
History of heart attack	0.01	1.80	1.15, 2.81
History of arterial hypertension	0.03	1.40	1.03, 1.89
Serum concentration of triglycerides (mmol/L)	<0.001	1.51	1.30, 1.75
Systolic blood pressure (mmHg)	0.01	1.01	1.01, 1.02
Number of meals taken daily	<0.001	1.46	1.25, 1.69
Religion Muslim versus non-Muslim	0.02	0.73	0.56, 0.94

## Discussion

In this population-based study in a typically ethically mixed urban and rural population of Russia, the prevalence of diabetes mellitus was 11.7% (95%CI: 11.89, 12.53) in the population aged 40+ years, with an awareness rate of 72.8% (95%CI: 69.0, 76.0). The prevalence of undiagnosed diabetes was 3.2% (95% CI: 2.7, 3.6): 3.6% (95% CI: 2.9, 4.4) in men and 2.8% (95% CI: 2.3, 3.4) in women. Among patients with diabetes, 59.1% (95% CI: 55.4, 62.8) received treatment for diabetes. Among those 406 diabetic individuals with diabetic therapy, 237 (58.5% (95%CI: 53.7, 63.3) individuals had a blood glucose concentration of lower than 7 mmol/L and were considered having adequate glycemic control. A higher prevalence of diabetes mellitus was associated with older age, higher body mass index, lower prevalence of vigorous daily work, positive history of arterial hypertension and including heart attacks, higher serum concentration of triglycerides, higher systolic blood pressure, higher number of meals taken daily, and non-Muslim religion (Tables 1–4).

The results of the present study cannot directly be compared with findings obtained in previous representative studies from Russia, since besides a first report on the prevalence of diabetes in a population of 912 inhabitants of the Siberian Krasnoyarsk region by Dogadin and colleagues, the present investigation is the first population-based investigation on diabetes in the European part of Russia. Measured by its size and ethnic composition, it is the first population-based assessment of the prevalence of diabetes in all Russia [5,14].

The diabetes prevalence of 11.7% in the population aged 40+ years in the present investigations compares well with a reported diabetes prevalence of 11.6% in the adult Chinese population in which, as in our study population, the prevalence of diabetes increased with older age and was higher in urban regions of habitation [15]. The figures for both, Russia and China, were similar to those reported recently for the U.S.A.., for which the National Health and Nutrition Examination Survey revealed an unadjusted prevalence of diabetes (as defined by a previous diagnosis of diabetes or a hemoglobin A1c level of ≥6.5% or a fasting plasma glucose level of ≥126 mg/dL) of 12.3% (95%CI: 10.8,14.1); among those with diabetes, 25.2% (95%CI: 21.1, 29.8) were undiagnosed [16]. As in our study, the prevalence of diabetes in the U.S.A. showed inter-ethnical differences, being lower in non-Hispanic white participants (11.3% (95%CI: 9.0, 14.1)) than in non-Hispanic black participants (21.8% (95%CI: 17.7, 26.7)), non-Hispanic Asian participants (20.6% (95%CI: 15.0, 27.6)), and Hispanic participants (22.6% (95%CI: 18.4, 27.5)).

It was remarkable that the awareness rate of diabetes mellitus in our study population from Russia was 72.8% (95%CI: 69.0, 76.0), a number similar to a general figure of awareness of 74.8% in the U.S.A., but considerably higher than the values reported for Hispanics/Latinos living in the U.S.A. with an awareness rate of 58.7%, values reported from China with a diabetes awareness rate of 30%, and numbers reported from India with a diabetes awareness rate of 25% [15–18]. The reason for the relatively high awareness rate of diabetes within the diabetic group in Russia may be that in accordance with the legislation of the Russian Federation, preventive medical examinations and additional survey methods are regularly conducted in health centers set up all over the Russian Federation (order of the Ministry of Health and Social Development of the Russian Federation of August 19, 2009 N 597n; http://base.garant.ru/12169847/). These examination conducted in these centers include assessment of the electrocardiogram, determination of blood pressure and calculation of the shoulder-ankle index, spirometry, measurement of the blood concentrations of total cholesterol and glucose, determination of carboxyhemoglobin and assessment of the concentration of carbon monoxide in the exhaled air (to identify heavy smokers), and analysis of cotinine in the urine (as measure of nicotine abuse). The health centers also include equipment for physical training and medical surveillance during the training sessions and teaching programs for a healthy lifestyle. The preventive medical examinations are conducted usually once every two years (https://www.rosminzdrav.ru/documents/6545-prikaz-minzdrava-rossii-1011n-ot-6-dekabrya-2012-g).

Among patients with diabetes in our study population, 59.1% (95% CI: 55.4, 62.8) received treatment for diabetes. This figure was higher than the figure reported from China where 25.8% (95% CI: 24.9, 26.8) of patients with diabetes received treatment for diabetes [13]. In a parallel manner, the percentage of treated diabetes patients with adequate glycemic control was higher in our study population from Russia (58.5%; 95%CI: 53.7, 63.3)) than in study populations from China in which 39.7% (95% CI: 37.6%, 41.8%) of treated diabetic patients had adequate glycemic control [15].

The prevalence of diabetes in our study increased with older age, higher body mass index, lower prevalence of vigorous daily work, positive history of arterial hypertension and cardiovascular diseases including heart attacks, higher serum concentration of triglycerides, higher systolic blood pressure, and higher number of meals taken daily (Tables 1–4). These associations were also found in numerous investigations and confirm the relationship between diabetes mellitus and its risk factors and sequels, such as lower degree of physical activity, more food consumed, obesity, older age, arterial hypertension, cardiovascular diseases, and elevated blood lipid concentrations. The association between higher diabetes prevalence and Russian ethnicity (non-Muslim religion) indicates additional cultural aspects potentially including inter-ethnic differences in alcohol consumption and diet.

Limitations of our study should be discussed. First, as for any population-based study the participation rate and the representativeness of the study population as compared to the population of the region or country the study is aiming at are critical. In our study, 80.5% of the eligible population participated in the survey so that a major bias in the inclusion of study participants may appear unlikely. The region of the study, a major city and a rural region in the Southern Russian republic of Bashkortostan West of the Ural Mountains was typical for the whole region of Southern Russia. Despite its relatively southern location, its continental climate with cold, harsh and long winters and warm to hot summers is relatively comparable with the continental climate in North-Western Russia and Central Russia. The multi-ethnic composition of our study population was typical for Southern Russia and showed as compared to North-Western Russia and Central Russia a lower percentage of Russians on the total population. To overcome this limitation, we assessed the prevalence of diabetes and its awareness rate in dependence of the ethnic background and found, that the diabetes prevalence was significantly (*P*<0.001) higher in the Russian group than in the non-Russian group (14.9% (95%CI: 12.9, 16.9) versus 10.0% (95%CI: 9.1, 10.9), while the awareness rate (*P* = 0.10) and the rate of adequate glycemic control (*P* = 0.08) did not differ markedly between the ethnic groups. The age and gender distribution in our study population was comparable to the results of the Russian census 2010. Second, we did not measure the percentage of glycosylated hemoglobin, so that the diagnosis of diabetes mellitus was based on the fasting blood concentration of glucose and the history of known diabetes.

In conclusion, in this Russian population, prevalence of diabetes in the adult population was similar to populations from other world regions. The awareness rate of diabetes was comparably high as was the rate of adequate diabetes therapy among those treated. As in other populations, the prevalence of diabetes increased with older age, higher body mass index, lower prevalence of vigorous daily work, positive history of arterial hypertension and cardiovascular diseases including heart attacks, higher serum concentration of triglycerides, higher systolic blood pressure, and higher number of meals taken daily.
